# PsicAP transdiagnostic protocol of group cognitive-behavioral training for emotional disorders in Dominican Republic: a randomized controlled trial protocol

**DOI:** 10.1186/s12888-023-04771-3

**Published:** 2023-05-24

**Authors:** Zoilo Emilio García-Batista, Luisa Marilia Cantisano-Guzmán, Kiero Guerra-Peña, Adriana Alvarez, Luciana Moretti, Antonio Cano-Vindel, Roger Muñoz-Navarro, Leonardo Adrián Medrano, Ricardo Araya Baltra

**Affiliations:** 1 Pontificia Universidad Católica Madre y Maestra, Santiago de los Caballeros, Dominican Republic; 2 Universidad Siglo 21, Córdoba, Argentina; 3 Complutense University of Madrid, Madrid, Spain; 4 University of Valencia, Valencia, Spain; 5 King’s College London, London, England; 6 Escuela de Psicología, Pontificia Universidad Católica Madre y Maestra, Av. Autopista Duarte Km. 1 1/2, Santiago, 51000 Dominican Republic

**Keywords:** Anxiety, Depression, Transdiagnostic treatment, Cognitive-behavioral techniques, PsicAP

## Abstract

Emotional disorders (ED) such as anxiety, depression and somatization are extremely prevalent disorders that can affect an individual’s quality of life and functionality. Primary Health Care (PHC) is the first place to identify most patients with these conditions. Mental health services in the Dominican Republic, as well as in Latin America and the Caribbean in general, are unable to provide appropriate care for most people with mental disorders. Using evidence-based treatment protocols is also crucial to make progress in helping people with ED. The PsicAP project is a group intervention that uses a transdiagnostic approach and is grounded in cognitive-behavioral techniques. The program is implemented in 7 group sessions, each lasting for one and a half hours. The program has been shown to be effective in reducing clinical symptoms, dysfunction, and in improving quality of life. It is also a non-time-intensive, low-cost treatment that is helpful for addressing EDs in a PHC context. The objective is to bring psychological treatments into PHC facilities of Dominican Republic, making them more accessible for a larger amount of the population.

## Background

Emotional disorders (ED) such as anxiety, depression and somatization are extremely prevalent disorders that can affect an individual’s quality of life and functionality [1]. The individuals who suffer from these disorders are not the only ones whose lives are affected; families and society in general also suffer due to the heavy burdens and socio-economic effects associated with EDs (2–3).

According to data from the World Health Organization [3], in 2015 more than 300 million people around the world suffered from depression (representing approximately 4.4% of the global population), while 264 million suffered from anxiety (equivalent to approximately 3.6% of the global population). After the onset of the COVID-19 pandemic, ED cases increased significantly due to factors such as social isolation, the death of close friends and family members, fear of infection and financial problems caused by the pandemic [4]. It is estimated that there may have been an additional 53.2 million cases of major depression around the world in 2020, as well as 76.2 million cases of anxiety disorders [5].

Specifically in the Dominican Republic, 464,164 people (4.7% of the country’s population) suffered from depressive disorders in 2015, while 570,312 (5.7% of the national population) suffered from anxiety disorders [3]. Following the onset of the pandemic, as in the rest of the world, there has been a significant increase in new cases of this type of ED in the country [6]. Despite this, the mental health centers and services in the Dominican Republic, as well as Latin America and the Caribbean in general, have not been bolstered in such a way where they can respond efficiently and effectively to the significant surge and increased prevalence of EDs. The lack of physical locations, financial resources, mental health professionals in Primary Health Care facilities or implementation of protocols to prevent and treat EDs continues to be a significant issue for the country [7].

If not addressed appropriately and in a timely manner, the chronicity, comorbidities and disabilities associated with EDs tend to increase [8]. Most patients do turn to PHC services for support and treatment. However, in these centers they are cared for by health professionals that are not appropriately trained for these types of psychological disorders [9]. As a result, patients are often under- or inappropriately diagnosed, leading to poor or inadequate treatment. Likewise, the lack of protocols and personnel trained to address mental health issues in PHC facilities leads to an additional outcome, where patients tend to be treated exclusively with psychotropic drugs. These medications do not solve the root of the problem or help patients manage their emotions, behaviors or thoughts, which can lead to chronic ED. Additionally, in the long term, psychotropic drugs can be addictive and toxic to the body, and can also cause an increase in traffic-related and domestic accidents (10–11).

Cognitive Behavioral Techniques (CBT) have shown that effective intervention strategies do exist to treat emotional disorders in both the child and youth populations, as well as in adults [12]. Using these strategies, individuals are able to identify their emotions, thoughts and behaviors while learning how to manage and restructure them. During the therapeutic process, the patient develops skills that enable them to recognize triggering situations and identify more adaptive behaviors that will help them develop strategies to face their difficulties more effectively [13–16]. Because of its effectiveness, CBT is the treatment of choice for various mental health care systems around the world [15].

Also, implementing CBT using a transdiagnostic model (approaching EDs as a unit with common characteristics and backgrounds) has been shown to be effective in reducing emotional symptoms [17–20] while also providing significant improvement in cost-effective and cost-utility relationships [21]. In fact, the transdiagnostic approach allows individuals with different but related emotional disorders to be grouped together [17], making it possible to save professional, economic and time-related resources when compared to individual and non-transdiagnostic therapies (13, 21–22).

In countries like England and Spain, it has been proposed that psychological therapy be included in PHC services in order to ease the aforementioned impacts and improve the mental health of the population [23]; furthermore, the cognitive-behavioral transdiagnostic protocol known as PsicAP was developed in Spain. A randomized controlled trial with 1,691 patients showed that group-administered transdiagnostic CBT was effective in reducing emotional symptoms [14]. Patients who participated in the PsicAP protocol in the intent-to-treat analyses showed a reduction in their anxiety (d = -0.65), depression (d = -0.58) and somatic symptoms (d = -0.40). These effects were also maintained after 12 months of follow-up.

In light of the favorable results obtained by the PsicAP protocol in Spain, the main purpose of this study is to replicate the same clinical trial in the Dominican Republic. Carrying out the PsicAP clinical trial in the Dominican Republic (ClinicalTrial.gov ID: NCT04642092) is a potentially beneficial alternative for country’s mental health in terms of improving life satisfaction, academic, occupational and social well-being, as well as overall quality of life for affected individuals [16]. In addition to the specific benefits for the patients, this improvement could also lead to positive effects for their families and society as a whole, while making better use of government health service financial and time resources, including those of PHC facilities (24–25).

## Objectives

### Main objective

The main objective is to undertake a randomized clinical trial to evaluate the effectiveness of a group cognitive-behavioral transdiagnostic intervention (PsicAP) compared to the typical PHC treatment for ED patients in the Dominican Republic at 3 months, and to explore if results are maintained over time (6 months). The main outcomes will include decreased symptoms of anxiety, depression and somatization, as well as remission of potential cases of ED.

### Secondary objectives

- To explore the efficacy of this new treatment compared to the traditional PHC treatments that are offered as a means of reducing dysfunction and improving quality of life, at 3 months.
- To explore the efficacy of the new treatment compared to traditional PHC treatments in reducing risk factors for cognitive distortions and emotional regulation strategies.
- To study the level of satisfaction with the treatments received.
- To develop a digital platform that includes content related to education, relaxation, cognitive/behavioral techniques and emotional regulation strategies for the general population and to provide additional training to therapists.

### Hypotheses

The hypotheses derived from the study objectives are that, in post-treatment and continuing through follow-up, patients who participate in the transdiagnostic cognitive-behavioral group treatment (PsicAP), as compared to the control group receiving typical treatment, will experience:

1. A more noticeable decrease in anxious, depressive and somatic symptoms.
2. Higher rates of remission when screening for possible anxiety, depressive and somatic disorders.
3. Better work, social, and domestic functioning.
4. A better quality of life.
5. A more noticeable decrease in risk factors for cognitive distortions and emotional regulation strategies.

### Study design

This is a randomized, controlled, two-armed clinical trial, with one arm consisting of the control group that will receive usual PHC treatment and another consisting of the experimental group receiving treatment according to PsicAP protocol. It should also be clarified that this is a simple blind trial, specifically in the fact that the evaluators are not the same psychologists as those giving the treatment. However, the study will not be blind in some respects, as patients can guess which group they belong to (since it is not possible to blind this condition), as will the therapists. Study participants will be assessed at 6 and 12 months after the seventh study session.

## Methods

### Study space

This is a multi-center trial carried out in 6 PHC centers; in most of these centers, psychological service is either non-existent or severely limited.

In order to improve mental health services in the country, the study protocol will initially be implemented in the following PHC centers in Santiago de los Caballeros, Dominican Republic: Dr. Sergio Bisonó Primary Health Center, El Guano Primary Health Center, Mamachen Primary Health Center, Barrio Obrero Community Center, Hato Mayor UNAP Center and Gurabo Diagnostic Center.

### Target population

The total intended sample that will be reached in this project is 300 Dominican adults aged 18 to 79. Adult patients from PHC centers who exhibit emotional symptoms (anxiety, depression or somatization) or probable mild to moderate anxiety (generalized or panic), mood or somatization disorders that have been assessed using the Patient Health Questionnaire (PHQ) are eligible (26–27) Specifically, those who meet the study inclusion criteria for the possible diagnosis of any of these disorders or have exceeded the cut-off score on any of the corresponding sub-scales will be part of the study: General Anxiety Disorder-7 (GAD-7) (generalized anxiety ≥ 10), PHQ-9 (depression ≥ 10), PHQ-15 (somatization ≥ 5), PHQ- Panic Disorder (PHQ-PD) (positive and panic ≥ 8, including the first 4 items). Patients who score ≥ 24 on the PHQ-9 or with suicidal ideation will be excluded and referred to a specialist.

All patients in PHC centers will have the same opportunity to be assessed in order to determine whether they could benefit from PsicAP psychological treatment. Those who meet the study inclusion criteria will be invited to be part of the study and, after accepting and signing the informed consent of their own free will, they will be distributed equally at random (1:1) between the control group (receiving the conventional treatment used in PHC facilities up to this point) and the experimental group (who will receive the PsicAP treatment).

## Inclusion and exclusion criteria

### Inclusion criteria

- Be between the ages of 18 and 79.
- Have anxiety, depression with a PHQ-9 intensity level that is ≥ 10, but not exceeding 24 points.
- Have a positive result for somatization (3 or more symptoms at maximum intensity).
- Not be receiving psychological treatment/psychotherapy.
- Be Dominican.
- Be able to read and speak Spanish at a basic level (elementary education).

### Exclusion criteria

- Have a diagnosis for a severe mental disorder such as bipolar disorder or a personality disorder.
- Have had a recent serious suicide attempt.
- Have a serious work, family or social disability.
- Have a severe emotional disorder (ex., comorbidities with substance abuse disorders) or other severe mood disorders.

## Interventions

### Control group treatment

The control group will receive the TAU provided by the primary care physician at the PC center. This treatment has been described in previous research as no treatment, standard treatment, pharmacological treatment and/or practical advice by the GP provided in routine care (Watts et al., 2015). In the Dominican Republic, as in other countries such as Spain (Cano-Vindel et al., 2016), psychotropic drugs are typically given to these patients to lessen the symptoms of the disorders, such as anti-anxiety medications and sleeping pills.

### Experimental group treatment

The experimental group receive evidence-based group cognitive-behavioral treatment. The protocol that will be applied is based on the protocol originally designed by the PsicAP group in Spain, led by Dr. Antonio Cano-Vindel. The protocol is based on 7 sessions, with each lasting 90 min. It will be offered once per week in groups of up to 8 people. The sessions are made up of the following elements:

- Psychoeducation that focuses on providing guidance about what emotions actually are as well as their purpose, on the difference between normality and disorders surrounding emotions, and other useful information to counteract some of the misconceptions that the population tends to have about emotions and EDs.
- Relaxation techniques focused on reducing the physical sensations of discomfort that can trigger ED, including abdominal breathing and progressive muscle relaxation.
- Training in cognitive restructuring to ensure that patients can identify cognitive distortions and pathological or irrational thoughts that they experience in everyday situations so they can replace them with more logical and healthy alternatives, thus making a change to the emotional intensity associated with distortions and thinking errors.
- Behavioral therapy, so that patients can identify their emotions and any flawed strategies they may have for regulating emotions and dysfunctional behaviors, replacing them with more functional strategies and repeating them until the individuals develop healthier and more adaptive habits to replace the old patterns.
- Relapse prevention, to maintain long-term change and ensure that participants can identify their risk situations and the alternatives they have for dealing with them. This includes practicing problem analysis skills from a cognitive-emotional perspective, Use the tools and practical exercises that have helped us. Continuing to practice, until it is overcome. Receiving an additional psychological appointment.

Table 1 outlines the resources used for each component.

**Table 1:** PsicAP Protocol components and resources

Therapeutic components	Resources
Psychoeducation	Information about emotions, anxiety, emotional disorders, cognitive-behavioral treatment components, cognitive errors, the relationship between thought and emotion, clarifications about existing misinformation surrounding emotions and emotional disorders.
Relaxation techniques	Abdominal breathing exercises and audio, progressive muscle relaxation and changes in focus.
Cognitive restructuring training	Ellis ABC model, information about rational and irrational thoughts, and exercises to detect and analyze irrational thoughts and distinguish them from irrational [sic] ones, identify and restructure thinking errors and provide positive self-instructions.
Behavioral therapy	Behavioral activation, exposure therapy and techniques, and training in social skills, assertiveness and problem solving.
Relapse prevention	Problem analysis skills from a cognitive-emotional perspective, tools and practical exercises that have helped us, continuing to practice, until it is overcome.

## Therapist training

Each PsicAP group is led by a therapist who is also working together with a co-therapist. All of the therapists who are assisting the patients in the Dominican Republic PsicAP project originally graduated with a Bachelor’s degree in psychology, which requires approximately 4 years of university training. One of the basic requirements for taking on a therapist role was also to have a master’s degree in clinical psychology or a related area. Some of the therapists also have a Ph.D. in psychology. Co-therapists have a degree in psychology but have not yet completed a master’s program. The latter received specialized training so they could carry out the initial and final project assessments and assist the therapists appropriately during the sessions.

In order to standardize the therapeutic model to be followed, two significant types of training were done: (1) each therapist had to take an online course on the in TEA platform, which consisted of 45 h of training to refresh themselves on how to manage anxiety disorders (course name: “Refresher on the treatment of anxiety disorders: a cognitive-emotional and transdiagnostic approach” [*“Actualización en el tratamiento de los trastornos de ansiedad: un enfoque cognitivo-emocional y transdiagnóstico”*]); (2) therapists and co-therapists met once a week for seven weeks in order to share the slides and resources from each of the sessions that would be given to the participants, in an effort to establish guidelines so that the therapy in each of the centers would be as homogeneous as possible.

## Measures

### Generalized anxiety disorder test-7 (GAD-7)

The GAD-7 uses 7 items to evaluate how often people have been bothered by symptoms of generalized anxiety in the past two weeks. Each answer option is scored from 0 to 3, with the scale ranging from 0 to 21. Scores ≥ 5, ≥10, and ≥ 15 represent absent, mild, moderate and severe anxiety levels, respectively. According to various studies, the Spanish versions of both the GAD-7 and its short version (GAD-2) have appropriate reliability and validity levels for screening generalized anxiety disorder [28] and were validated in the Spanish PsicAP project [29].

### Patient health questionnaire-9 (PHQ-9)

The PHQ-9 uses 9 items to ascertain how often individuals have demonstrated depressive symptoms in the past two weeks. Each answer option is scored from 0 to 3, so the total range goes from 0 to 27. Scores of 5, 10, 15, and 20 are the cut-off points for absence of depression, mild depression, moderate depression, moderately severe depression and severe depression, respectively. Studies have shown that the PHQ-9 tool has good validity, reliability (α = 0.799), sensitivity (0.72) and specificity (0.76) for screening depression in primary health care units [28].

### Patient health questionnaire-15 (PHQ-15)

The PHQ-15 uses 15 items to ascertain how often participants have been inconvenienced by somatic symptoms in the past four weeks. Each answer option is scored from 0 to 2, with the total thus ranging from 0 to 30. Scores of 5, 10 and 15 are the cut-off points for low, medium or high severity in terms of somatic symptoms, respectively.

### Patient health questionnaire - panic disorder (PHQ-PD)

The PHQ-PD section of the PHQ includes 15 items that make it possible to detect the presence of panic disorder. The first three questions include elements from the Diagnostic and Statistical Manual of Mental Disorders-IV classification system to review the history and frequency of panic attacks (i.e., “In the past 4 weeks, have you had an anxiety attack or sudden feeling of fear or panic?”). The next questions contain information related to the somatic symptoms of panic attacks (i.e., “shortness of breath” or “tingling or numbness in parts of the body”). There are two response categories: “no” (0 points) and “yes” (1 point). The Spanish version of this assessment will be used, which demonstrates appropriate psychometric properties and acceptable sensitivity and specificity levels [29].

Patients are considered to have a positive score in the PD section if all four parts of question 3 (a-d) are answered in the affirmative (4 points), along with [sic] four items from question 4 regarding somatic symptoms (4 additional points). However, since the primary objective of the screening tools is to achieve a high detection rate, we made several modifications to this evaluation algorithm in an attempt to increase the test’s sensitivity in detecting PD. The original assessment algorithm requires affirmative responses for the first four answers to question 3. In contrast, our modified algorithm requires an affirmative response to the first item (3a) and one affirmative response for items 3b, 3c or 3d [29].

### Sheehan disability inventory (SDI)

The original SDI is a 5-item self-reporting tool. In the first 3 items, the patient’s functionality at work or school and their social and family life are evaluated using visual scales, each with 10 points. Scores ≥ 5 on any of the three scales are associated with severe dysfunction. The last two items evaluate the number of lost or unproductive days due to the dysfunction. Santolaya Prego de Oliver et al. [30] found that a 4-item unifactorial model of this scale (SDI-4) had good and acceptable discriminatory and predictive validity respectively, and is an appropriate tool for evaluating disability due to depression in patients who attend Spanish PHC centers.

### WHO QoL BREF quality of life scale

WHOQoL-BREF is a self-report that assesses four areas of quality of life: physical health, psychological health, social relationships and environment. There are also two items that measure general health and quality of life, respectively. The total scale consists of 26 items, and participants respond based on how they felt in the past two weeks. The answers to each question are based on a 5-point Likert scale (1 = “not at all” to 5 = “completely”). The raw scores for each area can fall between 7 and 35 for physical health, 6–30 for psychological health, 3–15 for social relationships, and 8–40 for environment. The higher the score, the better the quality of life. This scale has demonstrated good levels of validity and reliability among populations in both Spain [31] and Latin America [32].

### EuroQol-5 D (EQ-5D)

EuroQoL is a simple tool that measures quality of life in terms of health, and it has two main parts. The first is a descriptive system where the participant indicates how they feel on the day of assessment in terms of their ability to move, their personal care, their daily activities, their pain and symptoms of anxiety or depression. The response for each item is based on three options: 1 (no problems), 2 (moderate problems), 3 (severe problems). The higher the score, the worse the quality of life. The second part is a visual analog scale where patients subjectively evaluate the state of their health on the day of assessment (ranging from 0 = worst state of health, to 100 = best state of health). This scale has proven to be a simple, appropriate and very useful tool for evaluating the quality of life of PHC patients [33].

### Ruminative response scale-brooding (RRS-B)

The original RSS contains 22 items, which patients use to indicate what they typically do when they feel sad or depressed. It uses a Likert scale with 4 response options (1 = never; 4 = always), and has two subscales: Brooding and Reflection. When assessing the convergent validity of the subscales, [34] found that the Brooding subscale has a stronger correlation with depressive symptomatology than Reflection. Only the Brooding subscale is used in this study, which was validated in the Spanish PsicAP project [35]. Consequently, the total score of this subscale ranges from 5 to 20; the higher the score, the higher the patient’s melancholic level.

### Penn state worry questionnaire-abbreviated (PSWQ-A)

The PSWQ is a 16-item questionnaire, and the Spanish version has demonstrated good reliability and validity [36]. It uses a 5-point Likert scale (1=“not at all typical of me” to 5=“very typical of me”), and the scale ranges from 16 to 80 points. Scores ≥ 16, ≥40, and ≥ 60 represent levels of low, moderate, or high concern, respectively. This study uses a shortened, 8-item version that assesses worry as an uncontrollable, generalized and excessive phenomenon, which was validated to be used in Spanish primary care patients [35].

### Inventory of cognitive activity in anxiety disorders-panic brief (IACTA-PB)

IACTA is a tool that evaluates cognitive activity in patients with various types of anxiety disorders including panic attacks (14 items), agoraphobia (14 items) and social phobia (20 items). Participants indicate how often they pay attention to cognitive distortions related to these disorders, scoring from 0 (almost never) to 4 (almost always). The higher the score, the higher the likelihood that the patient suffers from an anxiety disorder. This tool has demonstrated psychometric properties for screening this type of disorder [37]. A shortened, 5-item version will be used in this study to assess the thought errors of PHC patients [13]. The validity of this scale has been proven by the international PsicAP teams, and the intention is to evaluate it with the Dominican population by way of this project [35].

### Metacognitions questionnaire-negative beliefs (MCQ-NB)

Using 30 items, the MCQ-30 measures the differences in an individual’s metacognitive beliefs and their tendencies in regards to judgment and monitoring. Each response option is based on a 4-point Likert scale (from 1 = “do not agree” to 4 = “agree very much”). MCQ-30 scores range from 30 to 120 points. The higher the total score, the higher the pathological metacognitive activity. This scale has proven to be reliable and valid among various populations, including in Spain (38–39). In accordance with the Spanish PsicAP model, we will use the MCQ-NB, which contains only 6 items related to negative metacognitive beliefs about uncontrollability and danger [35].

### Cognitive distortions in emotional disorders questionnaire (CDTE)

The CDTE is a tool that evaluates how often certain cognitive distortions appear. It has 24 items, each with a response option ranging from 0 (almost never) to 4 (almost always), with the total score thus ranging from 0 to 96. The higher the score, the higher the levels of cognitive distortion. This questionnaire contains 4 scales that assess the main cognitive distortions that underly the most prevalent emotional disorders (generalized anxiety disorder, major depressive disorder, panic disorder and somatization disorder). These 24 items were tested in a sample of 1,753 participants. After an exploratory and confirmatory factor analysis, the questionnaire showed the presence of 4 factors, each with 4 items; sustained attentional bias (alpha = 0.96); split attentional bias (alpha = 0.95); magnification interpretive bias (alpha = 0.94); and catastrophizing interpretative bias (alpha = 0.96). High levels of discriminant validity were observed with the 4 emotional disorders (COR curve values; >0.80). These results are currently in the publication phase.

### Cognitive emotion regulation questionnaire-27 (CERQ-27)

The CERQ [40] evaluates nine different cognitive strategies that individuals use to regulate emotions when facing a negative event, which are: We will use the validated Spanish version of the CERQ-27, which contains three items per factor [41]. This allows to study the different strategies individually, and also to evaluate two second-order factors: maladaptive (rumination, catastrophizing, self-blame and other-blame) and adaptive strategies (positive refocusing, acceptance, positive reappraisal, refocus on planning, and putting into perspective).

### Treatment satisfaction survey

At the end of the treatment, all participants will assess their satisfaction with the treatment they received and will have the opportunity to rate it on a scale from 0 (“not at all satisfied”) to 10 (“completely satisfied”).

## Patient recruitment

The study will remain open from the beginning of the assessment until the necessary sample is completed.

The data collection process will be completed once it reaches 300 participants. Sample sizeG*Power3 program were used to determine sample size, which prioritizes statistical power. The effect size measures obtained in the Spanish PsicAP clinical trial were taken into consideration, which were moderate to strong. Based on these analyses, the sample size established for this study was 150 participants per study group (n = 300).

It should be noted that participants will only be included in the study after they have given their informed consent. Patients who do not meet the study inclusion criteria will be referred back to their PHC physician for alternative treatments.

## Randomization

The simple randomization method is used in this study to assign participants to one of the treatment groups (experimental or control). This kind of randomization is done using the Excel computer program in a 1:1 scheme, which makes it possible to obtain two homogeneous groups.

## Data collection

After signing the informed consent, the participants are registered in the PsicAP database using alphanumeric codes to keep their identities anonymous. Participants perform pre- and post-treatment assessments digitally using computerized systems. All pre-treatment assessments are done at the centers along with the co-therapist, using tablets that can connect to the internet. The data that is collected is stored on the Survey Monkey platform (surveymonkey.com). The same battery of tests applied at the beginning of the treatment is administered upon completion. Ideally this last battery is also completed at the center together with the therapist, in the same fashion as during the pre-treatment phase. However, the participant will be given the option to complete it on their mobile device via WhatsApp or email using a link provided by the co-therapist. In this case, the co-therapist has the right to contact the participants via telephone in order to remind them to complete the post-treatment and follow-up evaluations.

## Data analysis

Intention-to-treat (ITT) analysis will be performed. The ITT analysis will include all randomized patients in the groups to which they were randomly assigned. Analysis will take into account noncompliance, protocol deviations, dropouts, and anything else that happens after randomization. The two randomized groups will be compared in the treatment period; posttreatment; and at 3 and 6 months after treatment finalization. In addition, within-subject comparisons will be analyzed, contrasting pretreatment and posttreatment scores. The within-group and between-group differences will be examined using mixed-effect models, since these are considered more accurate than univariate and multivariate repeated measures of variance. Group differences will be analyzed after controlling for baseline levels, gender, age, and treatment center. Analysis will be carried out using SPSS version 21.0 (IBM Corp).

For primary outcomes, will consider group differences in anxiety (GAD-7), depression (PHQ-9), and somatic symptoms (PHQ-15) comparing baseline and post-treatment scores using a mixed-effect model. For secondary outcomes, will check between-group differences in the level of disability on daily life domains (work, social, and family life) and quality of life domains (physical, psychological, social, and environmental). The effect sizes of the treatment on primary and secondary outcomes (mean scores) will calculate by applying Morris’s d statistic, where the standardized effect of the treatment is defined as the difference between groups in mean PPC values, divided by the common standard deviation. In addition, a per-protocol (PP) analysis for primary and secondary outcomes will performer only in the patients who completed all follow-up measurements.

Finally, will calculate additional analyses: recovery, reliable recovery, and deterioration rates. The recovery index was defined as pre- treatment scores above the threshold on any of the three scales and below the threshold on all scales at either the post-treatment or 6-month follow-up assessment. The reliable recovery rate will calculate using a change score based on the standard deviation (S.D.). Thus, will use a change score of ≥5 for the GAD-7 and ≥6 for the PHQ-9 and the PHQ-15.

## Dissemination plan

PsicAP’s future goal is that, after examining its effectiveness and making the necessary changes in order to be the most effective for the Dominican population, the treatment protocol used will be shared with all PHC centers. This would mean that more clinical psychologists will be trained and that the Dominican Public Health System will create more jobs for them in primary health care facilities.

## Results

The project’s recruitment phase began on August 9, 2021 and will remain open until the target sample is reached. All post-treatment evaluations are expected to be completed by May 2023.

## Discussion

EDs such as anxiety and depression are among the most prevalent disorders affecting the world’s population. They significantly disrupt the quality of life of those who suffer from them, and in some cases can even lead to death. Likewise, these disorders also affect the relatives of these patients and the societies in which they operate, since they are one of the most debilitating types of disorders and can affect and individual’s work, academic and social capacities [3].

For several years, the consistent increase in the prevalence of EDs has already been worrying; however, with the arrival of the COVID-19 pandemic, the increase has been exponential. This, coupled with the shortage of clinical psychologists in PHC facilities, has had an increasingly adverse effect on the mental health of many people both in the Dominican Republic and around the world due to lack of adequate or timely treatment [5].

In the Dominican Republic, most PHC centers do not have professional staff who have been properly trained in the area of clinical psychology. As a result, the typical approach is that the general physician on call who sees the patient offers to prescribe psychotropic drugs. The patient is at a high risk of developing an addiction and dependence on these drugs, and they also do not treat the root of the problem, meaning that the patient will never adopt the necessary tools to fix or manage what is triggering the ED (10–11).

## Limitations

Despite all the strengths and benefits that the PsicAP project can offer to Dominican society, it is also necessary to mention its limitations, which have been recognized since its proposal. One of these is the fact that general PHC practitioners only take 7 to 10 min to examine patients, so they may not be able to use the initial screening test with individuals who come to them that could benefit from PsicAP. This makes it difficult to recruit participants. Likewise, another limitation is that not all patients who come to PHC facilities are aware of the importance of treating EDs and, despite suffering from them, refuse to accept psychological help. PHC facilities in the Dominican Republic tend to be frequented by individuals with a lower socioeconomic status who have not received sufficient information about mental health issues or their manifestations and consequences. Also, due to the fact that the individuals who go to PHC facilities tend to have lower education levels, many do not meet the criteria to be included in the study since one of the prerequisites is being able to read and write.

Another limitation for the study is the fact that the institutions where patients are being recruited were not chosen at random, but rather by convenience (the institutions that were included had agreed to support the project and provide spaces for its development).

One final limitation of the study is the high risk that participants may not attend all seven sessions required for the PsicAP treatment and may choose to leave at some point, which tends to be a typical limitation in longitudinal studies like this one [13]. One other limitation is the use of self-reports.

## Conclusions

If the results of this project are favorable as expected, the PsicAP project will prove to be a much more cost-effective resource for both the individual and society when compared to the cost, loss of time and decrease in quality of life associated with individual and private therapy. Each PHC center in the Dominican Republic will be able to implement a protocol based on evidence, a transdiagnostic approach and cognitive-behavioral foundations, with techniques that have proven to be more effective and efficient in treating EDs [17]. All of this will improve the mental health and quality of life of many Dominicans, while also optimizing the stability of institutions and society.

Likewise, the goal is that the benefits of the PsicAP program will help raise government awareness about the importance of opening up more work areas for clinical psychologists at the PHC level. The hope is also to raise awareness at the individual, institutional and state levels regarding the benefits of having proper mental health treatment at even the most primary level of health services. When mental illnesses are treated early on, in the beginning stages, patients have a much better prognosis [42].

## Data Availability

Data sharing for this manuscript is not applicable given that no datasets were generated during this specific study. However, this study is part of a larger project which will contain datasets. Information regarding the data of this project is available upon request from the corresponding author Z.E.

## List of abbreviations

CBT: Cognitive Behavioral Techniques
CDTE: Cognitive Distortions in Emotional Disorders Questionnaire
CERQ-27: Cognitive Emotion Regulation Questionnaire-27
CFI: comparative fit index
ED: emotional disorders
GAD-7: General Anxiety Disorder-7
IACTA-PB: Inventory of Cognitive Activity in Anxiety Disorders-Panic Brief
MCQ-NB: Metacognitions Questionnaire-Negative Beliefs
PSWQ-A: Penn State Worry Questionnaire-abbreviated
PHQ: Patient Health Questionnaire
PHQ-PD: PHQ- Panic Disorder
PHQ-9: Patient Health Questionnaire-9
PHQ-15: Patient Health Questionnaire-15
PHC: Primary Health Care
RMSEA: root mean square error of approximation
RRS-B: Ruminative Response Scale-Brooding
SDI: Sheehan Disability Inventory
TLI: Tucker-Lewis index
